# Memoirs of the Royal Academy of Medicine

**Published:** 1844-07

**Authors:** 


### Art. XIII.

*Mémoires de l'Académie Royale de Médecine.* Tome Dixième.—*Paris*, 1843. 4to, pp. 806.

*Memoirs of the Royal Academy of Medicine.* Vol. X.—*Paris*, 1843.

Whatever be the differences of opinion regarding the utility of such institutions as the Royal Academy of Medicine of Paris in advancing the interests of medical science, and developing the mental resources of those whose turn it has become to enter within their august pale, there can be no question in the mind of him before whom this very large volume lies, as to their charitable influence in providing work for needy printers. For ourselves, who have been accustomed to take rather a gloomy view of the direct benefits accruing from the discussions carried on by the members of such bodies—in which quickness of repartee too commonly passes for the evidence of superior attainment; in which, while the establishment of truth is the assumed object, its perversion is often the real and scarcely veiled aim; in which the dignity of the scientific inquirer is too often lost in the unseemly display of uneasy egotism; in which the vigour of philosophic disputation merges but too frequently in the noisy brawling of personal recrimination; in which all seem persuaded of the necessity of glorifying themselves, and making their neighbours look as small as possible; in which the point at issue remains at the end precisely as it was at the beginning, because those who possessed some little information regarding it have not condescended to instruct the *οι πολλοι* (*their* way of hugging themselves on their superior wisdom);—for ourselves, we say, taking, as we do, this view of the scientific and moral influence of the greater number of debating societies, it is matter of gratification that such small good even, as that mentioned, may arise from their existence. Was there ever, we would ask, a really valuable contribution, owing its production to such bodies, which would not have seen the light without them? While, on the other hand, look upon the sheets of trash annually generated by the vanity that regards the getting up of a volume in each twelve months as essential to the dignity of the society. To go no further, scan the contents of the unwieldy tome last issued by the Academy of Medicine, with its 750 pages; all that is in the remotest degree valuable here might have been readily conveyed in 150 pages—perhaps even this is too liberal an allowance.

First and foremost come three *éloges* of as many defunct members of the Academy, (whether the persons in question deserve such tribute to their memories is a secondary point, the main fact remains the same, "they were of *us*",) supplied by the indefatigable pen of M. Pariset, the Jules Janin of the medico-scientific arena. If the great and "*à jamais célèbre*" feuilletoniste has been known to make the humblest *ballerina* the celebrity of the town for at least a week—which at Paris means, in these matters, half a century at least—by dashing off a few sparkling lines in her praise, M. Pariset has done no less for many a small exhibitor in the Academy; only, unfortunately, the eulogised *savant* has not been alive to enjoy his brief hour of greatness. We are not disposed for an inquiry into the good taste or honesty of this system of indiscriminate laudation; and, however we might prove to our own satisfaction its deficiency in both, as long as the Academy has a secretary of M. Pariset's caliber, we fear there would be no chance of spreading our conviction to the Rue de Poitiers.

Some of the papers collected in this volume are medical, others surgical; we shall first anatomise the former.

I. M. Hippolyte Royer-Collard, Professor of *Hygiène* at the Faculty of Medicine, proposes to enlighten the Academy upon "Temperaments, considered in their relations to health." In an exordium startling, even among the Memoirs of the Academy of Medicine, for its verboseness and grandiloquence, M. Royer-Collard laments over the imperfection of existing "Hygiène" as a science,—signifies his conviction that, so long as its study is not closely bound up with a sound physiology, this imperfection must continue, and winds up by defining scientific "*Hygiène*" as "the profound knowledge of the relations of human life to *other* natural existences." A most lame and impotent conclusion, deficient even in verbal accuracy; for human life is not an existence in the sense given to this word by the definition.

The author considers first the actual state of knowledge on the subject of his paper; endeavours next to show that the current notions thereupon are false; and lastly, points out the path which he believes best calculated to lead to a more accurate and practical estimate of the various nature of temperament, and of the modifications, compatible with health, produced thereby.

Health is not, we are told, an absolute unvarying state, an entity always identical; it varies in individuals, and this difference has been referred to and expressed by the phrase peculiarity of constitution. This peculiarity of constitution has naturally been made the subject of investigation, and observers led to refer it to this or that special condition of organic structure. Upon this special condition (the predominance of some organic system or fluid of the body) has been founded, as is well known, the distinction of temperaments, *nervous*, *lymphatic*, *bilious*, &c. The author hesitates not to denounce this doctrine as more or less completely false in all its parts, conceived, as it was, under the influence of the old humoral doctrines or an equally erroneous solidism. Take, for example, the sanguineous temperament: Is the quantity of blood here unduly great? Its celerity of circulation above the ordinary standard? Are the heart and great vessels more vigorously endowed than in other temperaments? Is respiration more active and the lungs more fully developed? All these questions are, according to M. Royer-Collard, commonly answered in the affirmative, whereas he would give them either a very decided negative or maintain the affirmative to be unproved. The lymphatic, bilious, athletic or muscular, and nervous temperaments are similarly passed in review in respect of their commonly admitted causes. In all, the result is the same; the author attempts to show the error of accepted doctrines. And he succeeds, truth to say, in the easy task; all has been admitted on the grounds of plausibility and facility. The matter required reexamination; and though the process might have been less prolixly and less pompously performed than by this "candidate for the honours of the Academy" (possibly, aspirants have learned to trust to the avoirdupois, as much as to any other kind of weight of their papers), it is perhaps well that even he has been found willing to undertake it.

No, says M. Royer-Collard, if temperament be a *general* state of the economy, it follows that its source must be sought in something similarly general in the economy, not in an "isolated fluid" as the bile, or a special organ, as the liver or muscles. Its cause then must be sought in the two "principles that animate all the parts of the organized machine, ...... the blood and nervous action." Hence he concludes that, "not the cause, but the *characters* of temperaments should be studied in the various manifestations presented by those two principles." And so "*voilà le premier pas fait*," according to our philosopher.

Commencing with the blood, the writer admits the mean relative proportions of the main ingredients obtained by the inquiries of recent experimentalists; insists upon the tolerable wideness of the limits within which those proportions may oscillate, and yet health be preserved; remarks upon the increase of the colouring corpuscles which Andral has shown to attend the state of plethora, as also the diminution of those particles found in individuals of lymphatic temperament. The organic materials of the blood generally follow the same law of increase and decrease as the corpuscles; the ratio of these different organic materials to each other varies, and hereby hang sundry variations in temperament.

These are the views of M. Royer-Collard,—at least a specimen of them, a brick of his Babel. Those interested in such edifices must examine the entire structure for themselves; we have given it as much space as it merits.

II. M. Mélier investigates with a philosophic spirit the important medico-political question of the influence of a varying state of the price of provisions on general disease and mortality, in a paper entitled "*Etudes sur les Subsistances envisagées dans leurs rapports avec les maladies et la mortalité.*" The statistical documents he has collected, and the tables into which his figures are thrown, bear out the following inferences, to the enumeration of which we must for the present limit our notice of the paper. "1, The mortality of a country is influenced by the price of corn and bread; 2, this influence was extremely marked formerly; 3, it is less so at present; 4, the diminution of this influence has been gradual; 5, various causes have contributed to this result; 6, the cultivation of the potato is one of the chief of these; 7, less bread appears to be consumed at the present day than formerly; 8, the consumption has not been calculated accurately, but its amount appears to be on the decrease; [these two propositions refer, as far as the author is concerned, to France only;] 9, the question now considered is one of morality as well as of hygiène; for it is demonstrated that crime increases with the dearness of provisions." It is inferrible too, the author considers from his inquiries, that in a well organized state of society, provisions tend constantly to increase in abundance; this tendency, in France at least, is more marked than that of the population to increase,—"a powerful argument against the theory of Malthus."

III. *Sur la valeur de l'examen microscopique du lait dans le choix d'une nourrice.* Par M. Alph. Devergie. Since the announcements made some years ago by M. Donné, on the subject of the micrology of milk, and especially since that gentleman was intrusted with the very momentous office of choosing, microscope in hand, a wet-nurse for the Count of Paris, it has been a sort of "*passion*" among the very excitable Parisians to study the milk of all manners of women with that instrument. M. Devergie, stricken with the reigning enthusiasm (and naturally enough, for it appears that in 1838 and 1839,* just at the period when M. Donné's discoveries shone with all the eclat of novelty, he was invested with the office of Director of Nurses,) set to work, like the rest of the world, "having first made himself sufficiently skilful in the use of the instrument" to undertake the appreciation of M. Donné's statements. In the course of seven months he collected 172 cases, taken without selection, noting in every instance the following particulars: 1, the date of observation; 2, the nurse's name; 3, her age; 4, her temperament; 5, her consti-

* The author does not state whether this was the time when the very ingenious observer above named made his remarkable discovery that the conversion of blood-corpuscle into pus-corpuscles is producible by the admixture of pus and blood out of the body! Such achievements should not be dateless.

tution: 6, the colour of her hair; 7, that of her skin; 8, the size and various physical characters of the breasts; 9, the width of the chest; 10, the state of the teeth; 11, the length of time elapsed since the cessation of lactation; 12, the age of the milk; 13, the microscopical examination of the milk since the cessation of lactation; 14, the microscopical examination of the milk subsequently to the recommencement of lactation."

In respect of its globular character, the author considers that human milk is of three kinds: 1, large-globuled milk; 2, small-globuled, generally "pulverulent" milk; 3, milk of medium-sized globules. Not that any given specimens of milk will contain one or other of these kinds of globules to the complete exclusion of the others, but that there will be a sufficient predominance of one or the other to distinguish it. The large-globuled variety is the most nutritive; and the nutritive properties of the others are proportional to the size of their globules. This statement results from a comparison of the strength and bulk of infants, with the microscopical characters of the milk used to feed them, and also from comparing the milk with the general health and vigour of the nurse. The results were confirmed by examinations of cow's milk. The microscope then will enable us to determine in doubtful cases whether a given milk be of the strong or weak class, and will guide the physician in the choice of a nurse, wherever the question turns upon the advisability of one or other of these kinds.

Milks differ not only in respect of the size of their globules, but also of the abundance of these; high or low amount of the globules signifies richness or poorness of the milk generally: the author uses an arbitrary scale, running from the number five to forty to express variable degrees of these qualities. The milk furnished by the two breasts is not always the same; a difference may exist in respect of the number or size of the globules,—the former much more commonly than the latter.

Out of 100 nurses, 17 had the large-globuled variety of milk; the majority (10) of these persons were of sanguineous or sanguineo-lymphatic temperament. The large size of the globules does not always entail a maximum amount of number of these, in other words, of richness of the fluid. Thus, in these 17 nurses, the degree of richness is expressed by the author as follows:

| | | | |
|---|---|---|---|
| In 4 cases, | the richness | equalled | 10 |
| 3 | ,, | ,, | 15 |
| 3 | ,, | ,, | 20 |
| 3 | ,, | ,, | 25 |
| 4 | ,, | ,, | 30 |

In 10 of these cases an increase in richness took place during lactation, and

| | | | |
|---|---|---|---|
| In 5 cases, | the richness | equalled | 40 |
| 2 | ,, | ,, | 35 |
| 1 | ,, | ,, | 30 |
| 2 | ,, | ,, | 25 |

This state of the milk does not always coincide with the external appearances of a good nurse; 4 of the 17 individuals referred to were thin, puny, and with prominent clavicles. It is, however, true and well known, independently of microscopical examination, that puny women sometimes make better nurses than those exhibiting a far more favorable physical condition.

The small-globuled or pulverulent milk existed in 22 of 100 nurses; here the temperament of the individual varies, as in the case of the last variety, and in about the same proportion. Of 23 cases of small-globuled milk,

| | | |
|---|---|---|
| In 9 | the richness equalled | 5 |
| 2 | ,, ,, | 10 |
| 5 | ,, ,, | 15 |
| 5 | ,, ,, | 20 |
| 1 | ,, ,, | 25 |
| 1 | ,, ,, | 30 |

And, as respects the increase of richness produced by lactation, the author found that among 17 nurses,

| | | |
|---|---|---|
| In 4 | cases, it reached | 40 |
| 1 | ,, | 35 |
| 5 | ,, | 30 |
| 2 | ,, | 25 |
| 1 | ,, | 20 |
| 1 | ,, | 15 |
| 1 | ,, | 10 |
| 2 | no change. | |

Whence, comparing these results with those having reference to the large-globuled variety, it follows that the small-globuled is generally poorer in quality, and generally less capable of acquiring increased richness from the resumption of lactation. So that suppose a woman presents herself as a candidate for a nurseship, not having given the breast for some few days, there is less chance of the milk becoming eventually rich, if it present small than if it contain large globules. Milk of medium-sized globules is the kind the most commonly observed; 61 of the 100 women furnished this description.

A woman may present differences in the milk of the two breasts, as already mentioned; these differences of richness and size of the globules are often dependent upon the habit nurses have of suckling with one breast rather than the other, so much so that when the difference is very great, we may be certain that the individual uses only one breast.

Increased richness of the milk is an almost constant result of resumption of lactation after temporary interruption. The age of the milk has no influence in increasing the number or size of the globules: nor does the age of the nurse influence them either. Very large and very small breasts furnish milk of the least favorable qualities; the colour of the hair has no obvious connexion with varieties in the microscopical characters of the milk, and the same is true of the development of chest in different individuals.

Morbid changes in the microscopical characters of the fluid are rare. The most common consists in the agglomeration of globules, a state which may disappear under the influence of lactation. Important changes in colour—the milk may become blue or green to a marked degree—occur without any concomitant modification in the microscopical appearances. Yet the physiological character of the fluid is materially altered, so that the infant's health suffers at once; the microscope, then, is not the sole necessary guide in the question of choosing a nurse.

A good nurse, the author concludes, should be from twenty-five to thirty years old, strong in constitution, full-chested, of sanguineo-lymphatic temperament, brown-haired, having white healthy teeth, and well coloured lips. She should have pyriform breasts, with well-formed nipples, and without too much development of veins. Round prominent breasts with large veins, and broad areolæ are comparatively very inferior. The milk drawn into a spoon should be white with a slight bluish tint, its taste saccharine; it should not be too thick. These facts ascertained, the microscope comes into play, and demonstrates that the milk is or is not altered in the character of its globules, that it is more or less rich in globules, that its globules are more or less large. But the microscope cannot prove that a given milk should be preferred to another in a given case, because the health of the infant depends entirely upon the assimilation of the milk by the digestive organs, and the instrument gives no information upon this head. But the microscope may be of great utility in aiding the physician in the choice of a new nurse in cases where a previous one has furnished milk disagreeing with the infant; whatever microscopical peculiarity exists in the globules of the latter, will of course be studiously avoided in the milk recommended for new trial.

This is a paper of interest and utility.

IV. *Etude de la phthisie à la Martinique.* Par le Dr. E. Rufz. M. Rufz having returned to his native island, after a series of scholastic triumphs at Paris, (in the last of these, the concours for some vacant places of Professor Agrégé at the faculty, his was probably the most brilliant exhibition made for years, even in that arena of display,) proves himself not forgetful of the scientific pursuits of his earlier years. The Academy of Medicine signifies its desire for information respecting the frequency and peculiarities of pulmonary consumption in warm climates, and the ex-professor agrégé responds to the desire by a paper describing the malady as it prevails at Martinique.

Pulmonary affections, with the exception of phthisis, appear to be singularly rare. The writer has met with but three cases of pneumonia in five years. Chronic bronchitis even among old subjects is similarly uncommon; gastro-intestinal affections cut off the advanced in age. This infrequency of other pulmonary diseases facilitates the diagnosis of phthisis.

The author practises at Saint-Pierre and its environs, in a district containing, according to the official census of 1835, a population of 17,000. Some other practitioners divide the care of this population among them. The following is an approximate view of the frequency with which phthisis occurred in M. Rufz's practice.

| | | | | | |
|---|---|---|---|---|---|
| In 1836, | among about | 600 | patients, | 40 | were noted as phthisical. |
| 1837 | ,, | 378 | ,, | 21 | ,, |
| 1838 | ,, | 445 | ,, | 35 | ,, |
| 1839 | ,, | 531 | ,, | 27 | ,, |

Thus, out of 1954 patients, 123 were tuberculous; in other words, about 13 per cent. Phthisis is the most common chronic disease in the island. Scrofulous allied affections are nevertheless comparatively rare; the author has seen but one or two cases of white swelling; no example of tuberculous caries of the vertebræ; glandular swellings also are uncommon, and but two examples of tuberculous meningitis have fallen under his notice. Phthisis is excessively rare in the child at Martinique; the author having encountered pulmonary tubercles but twice in all the post-mortem examinations made of children,—he does not say, approximatively even, how many these were. But it follows from this, that all the author's statements are to be understood as applicable to the adult only.

One hundred and eight of the 123 phthisical patients observed by the author had been followed by him in such manner that at the time he wrote he knew whether they were living or dead; 55, or somewhat more than half were dead. Nine only of these subjects were examined anatomically. The thoracic lesions were the same in these subjects as in European victims of the disease; but it is remarkable that in seven cases hemoptysis was the immediate cause of death, whereas in these countries such a *termination* of the malady is extremely rare. In none of these instances was the author able to discover a ruptured vessel of any caliber, to account for the extravasation of blood.

Tuberculization of other organs besides the lungs is much less frequent at Martinique than with us. In 2 only of 9 cases did the author detect tuberculous ulceration of the intestines, a rarity testified too also by the infrequency of colliquative diarrhea in the phthisis of the island. The mesenteric glands are rarely tuberculous. The liver was fatty in 1 only of the 9 subjects; as we have had occasion to state in this Journal, fatty liver is very rare in this country also, but in France M. Louis met with it in about one third of his post-mortem examinations.

Of 53 fatal cases, 1 only would be regarded as acute in its course; all the others lasted at the least three months, many of them several years, as appears from the subjoined table.

| | | | |
|---|---|---|---|
| 3 | terminated by death | in | 3 months. |
| 11 | „ | from | 3 to 12 months. |
| 7 | „ | „ | 12 to 18 „ |
| 8 | „ | after | 3 years. |
| 1 | „ | „ | 15 „ |
| 3 | „ | „ | 30 „ |
| 13 | „ | „ | several months, *i. e.* from 6 to 18. |

The course of the disease in respect of duration seems to be very much the same, consequently, as with ourselves.

M. Rufz proceeds to the examination of some of the symptoms of phthisis. The consideration of hemoptysis leads him to inquire whether that symptom when severe, is as indubitably significant of the existence of phthisis in Martinique, as it has been shown to be by M. Louis in France. In the course of four years, 73 persons, who had had hemoptysis, fell under his observation. Of these 73, 39 (27 females, 12 males,) died of phthisis; 34 were still living, when the author wrote. Among these 34 persons, 19 were women, and 15 men. Of the men, 10 were in the enjoyment of good health, free from evidence of any kind of phthisis or cardiac disease, and the author is certain that the hemoptysis in them was not an effect of external violence or accident; the other 5 are of weakly constitution, cough and exhibit other signs of phthisis. Of the 19 females, 12 could not be considered phthisical, 7 might be so considered. Hence 51 of 73 persons who had had hemoptysis are phthisical.

The author considers that these results do not differ much from those which led M. Louis to regard hemoptysis as an almost unfailing sign of tuberculous disease. They differ less, we believe, than might on first sight appear; but did they swerve infinitely more from them than they do, the fact would supply no shadow of an argument against M. Louis' proposition, limited as it was to France and countries of similar temperature. If there be a practical point in medicine well established, it is, in our minds, the diagnostic signification in these countries of copious hemoptysis, as pointed out by M. Louis.

M. Rufz found diarrhea exceedingly rare in the phthisical inhabitants of Martinique: this is a remarkable point of difference between the diseases here and in that island.

M. Rufz has employed emetics in the treatment of the disease with the following results: In 3 out of 20 cases, he was obliged to give up their use, simply because they fatigued the patients, and not from any other cause. In the 17 other cases, their employment was almost always followed by relief of cough, increased freedom of respiration, and improvement of appetite. He never observed diarrhea follow their employment, nor was the number of patients suffering from symptoms of softening of the stomach considerable. In two cases emetics arrested hemoptysis which venesection had failed to control.

The author concludes from his own observation that, *except* age, sex, constitution, habitation of large cities, and hereditary influence, all presumed causes of phthisis have in reality a very secondary influence. We know not where M. Rufz has obtained his assured belief of the very essential influence of some of those agencies to which he ascribes such power.

Of the various races inhabiting the Antillas, Europeans and Africans are less subject to phthisis than creoles; and among the creoles, the white and mulatto are more prone to the disease than the negro. M. Rufz further infers that the influence of the climate of the Antillas on patients coming from Europe, in a state of declared phthisis, is not deducible from his own experience; but that, on the other hand, the removal from those islands to Europe of phthisical persons, is extremely pernicious.

*On epidemic cerebro-rachidian meningitis and encephalo-meningitis.* By M. Rollet. An epidemic disease, as rapid in its course as destructive in its results, has, it appears, for some years past, ravaged the garrisons of Versailles, Lyons, Bayonne, Groit, Metz, Strasbourg, &c. Nor does it appear to have been altogether confined to the military: the civil population of the environs suffered in 1838 under an epidemic visitation, as described by Drs. Lamothes and Lespès, closely analogous to that affecting the garrisoned soldiers of the towns enumerated. This epidemic M. Rollet regards as an encephalitic affection, in which he follows other writers; while, in respect of the precise mode of change and sequence of affections of the membranes and the cerebral substance itself, he differs from them. We shall, as briefly as we possibly can, give some notice of the observations of this writer.

In respect of predisposing causes, extreme youth of the soldier, his having recently joined the service, and being unaccustomed to military exercise appear to be the most obvious. Prolonged exposure to the sun, with or without subsequent chill, but abôve all violent exercise followed by chill, appear to have acted most frequently as immediate causes. Miasmatic influences, and those productive of infection, are regarded by the author as completely unconnected with the development of the malady.

The symptoms are regarded as belonging to three separate periods; and this both in the cases where the disease is simple meningitis and when the encephalon is likewise implicated. These symptoms are very carefully described; but the enumeration occupies too much space to admit of its extraction, nor could it be condensed to any useful purpose.

The morbid changes detected in the brain, cerebellum, spinal marrow, and meninges perfectly justify the nomenclature adopted for the disease by M. Rollet: Injection of the membranes, with effusion of red-coloured serosity, pus in the pia mater, softening of the medullary substance of both brain and cord.

The treatment recommended for the cases of simple meningitis is essentially the antiphlogistic, accompanied with derivatives to the extremities, purgatives, cold applications to the head, &c. This method of treatment appears to have invariably succeeded, and no fatal case occurred.

But when the substance of the brain and cord is implicated, the case is infinitely more dangerous. Occasionally, when the patient is first seen, he is in a state of collapse, such as renders venesection inapplicable: should, however, a state of reaction exist, bleeding ought to be copiously employed. The great point upon which the writer lays most stress is this: When patients are in the state of collapse — the limbs relaxed, the sensibility completely gone, the skin cold, and the pulse filiform—bleeding is contraindicated, until reaction towards the surface shall have been effected. Now the ordinary modes of exciting such action—blisters, sinapisms, ammoniacal ointment—fail; the skin is completely insensible to these irritant agents. This M. Rollet had learned by his own experience, as well as by the utter failure of such means as employed by others in a similar conjuncture. He determined to try still more energetic means of exciting the skin—none other than the application of the actual cautery along the spine. While applying this, sinapisms are placed on the feet, two large surfaces vesicated on the thighs with ammoniacal ointment, if there be room at the nucha some blood is drawn there by cupping, and a purgative enema administered.

The scarifications excite no manifestations of sensibility on the part of the patients; the first applications of the hot irons are similarly without effect, and it is only towards the fourth or fifth that slight muscular movements, or, in some instances, cries on the part of the patient give evidence that he feels. All of them immediately relapsed into their previous comatose state, no matter what slight demonstration of suffering they may have made. An hour or two after the cauterization, reaction commences; when it is established, general and local bleeding must be had recourse to, to an amount proportional to the strength of the individual, and associated with the other means of treatment already enumerated. Numerous subsidiary points are considered with due care by the author: for these, as well as for the answers adduced in reply to several objections which have been urged against the method of treatment generally, we must refer to the original.

The numerical results of all the cases treated are as follows:

Of 14 cases of meningitis, not one terminated fatally.
Of 14 cases of encephalo-meningitis,

| | | |
|---|---|---|
| 4 treated on the old plan . . . . . | 4 *died,* | 0 *recovered.* |
| 10 treated on the author's plan . . . | 4 | 6 |

Of the 4 who died of these latter 10 patients, 2 were so far advanced when admitted as to be beyond the reach of influence by any treatment whatsoever; another fell a victim to a relapse, having previously appeared in a favorable way to recovery.

Dr. H. Parrot communicates a *History of the epidemic of miliary sweating-sickness, which ravaged the department of Dordogne in* 1841. This is an elaborate and very admirable history of an epidemic of this disease, which affected one seventh of the population of the districts in which it appeared, and destroyed the lives of one thirteenth of those seized. The admirable topographical descriptions of the regions where the disease prevails, and the accurate investigation of its causes, are particularly worthy of attention. But the paper sins, like almost all its fellows, by its unconscionable prolixity, and is not of sufficient interest for the general medical reader to justify us in devoting any space to its analysis.

M. Mélier insists, in an essay on *Intermittent affections, in which the period of intermission is short*, on the importance of quinine as a remedial agent. Thus, in encephalic irritations of infants, with convulsions, in severe cases of hiccup, (so severe, that the author thought they must—as has been the fact in some instances on record—prove fatal, unless speedily relieved;) in uterine neuralgic pains, in neuralgia of various nerves, he has prescribed sulphate of quinine with almost magical success.

The fundamental point in the paper is the author's position, that the quotidian, the tertian, the quartan, &c. are not the only types of *intermittent* diseases, but that there are others to be regarded as of the same category, wherein the period of intermission is extremely short. In all such affections quinine is absolutely indicated. The paper is well worth perusal.

A learned, ingenious, and elaborate essay on the *Seat, intricate nature, symptoms, and diagnosis of hypochondria* follows, from the pen of M. F. Michéa. We can only, however, refer to it, as deserving the study of persons interested in the subject of which it treats.

M. Prus furnishes a memoir on *Pulmonary emphysema, considered as a cause of death.* The author commences by a brief summary of the position established by some of the chief writers on emphysema, closing this survey by an affirmation, that numerous points remain still to be elucidated in the history of this morbid state. The principal questions which he conceives require examination are: What is the disease to which the name of pulmonary emphysema is given? Does pulmonary emphysema consist in the dilatation of the pulmonary vesicles, or in distension of the intervesicular cellular tissue with air? Is asthma a distinct disease from pulmonary emphysema? What are the causes of pulmonary emphysema? Have the modes of prognosis and consequences of this morbid state been sufficiently studied? What should be our prognosis in cases of pulmonary emphysema? Is the affection susceptible of cure? May it of itself cause death? May it determine disease of the heart, more particularly of the right ventricle? What are the affections of which it increases the danger? What has been the influence of the study of pulmonary emphysema on the treatment of asthma?

We have enumerated these queries because we agree with their author in considering that the points they refer to require, every one of them, additional investigation. M. Prus limits himself, in the paper before us, to the subject of the prognosis of the affection, incidentally examining the question of the intimate seat of the disease.

The great object of this writer is to show that Laennec, Louis, and others, who regard uncomplicated pulmonary emphysema as an affection which does not directly destroy the patient's existence, are seriously in error. In M. Prus' estimation, the disease is one of great danger in a certain number of cases, while he admits multitudes are on record, and present themselves in daily practice, exhibiting on the other hand its comparatively frequent harmlessness. But this difference of opinion is more apparent than real, and arises altogether from (as we believe it to be) the serious error committed by M. Prus in regarding all cases, known symptomatically as examples of emphysema, to be constituted anatomically by effusion of air into the intervesicular and interlobular cellular tissue of the lungs. That this latter affection is occasionally a most serious one, and that it has occasionally proved the cause of sudden, and even instantaneous death, is a fact which has been known and recognized by pathologists for years. We find several examples referred to in illustration of the fact in the article Emphysema, in the 'Cyclopædia of Practical Surgery,' (vol. ii, p. 77,) by Dr. Walshe. The whole point at issue with M. Prus, (who adds eight cases of the kind just now referred to in the memoir before us,) lies then in a nutshell,—the simple question is whether in all cases of Laennec's emphysema, effusion of air into the cellular membrane is a constant existence. Now we hesitate not to affirm that M. Prus fails to demonstrate this. He simply asserts that in all cases air exists between the cells, but he by no means proves such to be the fact. And in stating the opinions of his predecessors, he commits the inaccuracy of leading his readers to suppose that they maintain dilatation of the vesicles to constitute the whole amount of morbid change existing in these cases. On the contrary, it has been taught by many of them that atrophy or hypertrophy of the walls of the cells, attended in some cases with destruction of those walls, exists at the same time. Now we should be led, by *à priori* reflection, to imagine that interlobular and some general subcutaneous emphysema would prove a frequent accompaniment of dilatation of the cells, inasmuch as it is frequently associated with rupture of their walls. But experience proves it (notwithstanding the affirmations of M. Prus to the contrary) to be singularly rare. Now the cause of this is a question of no little interest, and it appears to us to be plausibly pointed out in the following passage taken from the article by Dr. Walshe just referred to. (p. 77.) "Effusion of air into the cellular membrane is, however, a very rare consequence of an opening effected in the vesicles by the slow process of atrophy and distension. This rarity is probably owing to the following circumstances. In the majority of instances, perforations of the cells, as we have had frequent opportunities of ascertaining, open, not into the investing cellular tissues, but into the adjoining vesicles, the walls of which coalesce with their own. Secondly, the parietes of some vesicles are hypertrophous, and hence by obstructing the surrounding interstices, contribute with the natural density of the cellular membrane, to impede the circulation of any bubble of air that may escape from the cells; while freedom of circulation is the more necessary for the productions of subcutaneous emphysema, because vesicular emphysema is commonly most highly developed at a considerable distance from the root of the lung, namely, at its anterior border."

*Experiments and observations on the poisonous properties of disulphate of quinine.* By M. F. Melier. This paper, to which the author's apprehension, that the dangerously large doses of quinine recently recommended in the treatment of acute rheumatism by M. Briquet, might be generally adopted, has given rise, is full of practical interest. M. Briquet alleged, be it remembered in the first place, that he had cured acute articular rheumatism, accompanied with violent pain, swelling, redness, fever, &c., in two or three days with disulphate of quinine, administered to the amount of six grammes [about a drachm and a half] daily. Whether this system of treatment does or does not cut short, with very surprising rapidity, many cases of rheumatism, (and indeed we may admit that it actually has done so,) is not the present question ; but that it is a most dangerous mode of treatment is rendered obvious by the simple statement that it killed a fair number of rheumatic patients during the short period of its vogue at Paris.

First, as respects the experiments of M. Mélier on dogs, the general result is that the salt appears to prove fatal through its influence on the blood. It is more rapidly fatal, and in smaller doses if given dissolved in sulphuric acid than in powder, and when the stomach is empty than after taking food.

In the human subject the ill effects distinctly observed to follow the immoderate use of quinine have been, death ; delirium and coma ; pneumonic symptoms ; hematuria ; amaurosis ; deafness ; obstinate gastralgia ; diarrhea ; epileptiform phenomena ; paralysis. All these conditions are illustrated by reference to cases ; and a most melancholy and remarkable one added, in which a medical practitioner, blindly confiding in the curative powers of the drug, ended in poisoning his wife with enormous doses of it, and very nearly crowned his folly by destroying his own life in the same way ; he remained for a long time both blind and deaf after his recovery.

